# Notes and News

**Published:** 1889-12-28

**Authors:** 


### Notes and News.

Collected and Communicated.

Some notice must be taken here of the great progress in the hospital world which is especially noticeable in last year's annals. Amongst the chief new buildings which have been opened are Blackheath Cottage Hospital, Bournemouth Royal Victoria Hospital, Derbyshire Convalescent Home, Drumshoreland Infectious Hospital, North Devon Convalescent Home, Epsom Cottage Hospital, Glossop Jubilee Hospital, Gloucester Eye, Ear, and Throat Hospital, Harrogate Bath Hospital, Heathcote Infectious Hospital, Hereford Victoria Eye and Ear Hospital, King's Norton Infectious Diseases Hospital, Newcastle College of Medicine, Oxford Eye Hospital, Tynemouth Infirmary, Tolworth Isolation Hospital, Turton Convalescent Home, and Wakefield Isolation Hospital.

Amongst the chief extensions to existing buildings have been a new wing to Birmingham Orthopœdic Hospital, two wards to Brighton Throat and Ear Dispensary, nurses' home to Birmingham Queen's Hospital, Jubilee wing to Dover Hospital, new wing to the Eastbourne Princess Alice Memorial Hospital, children's ward to Grantham Hospital, new storey to the clinical side of Leith Hospital, Lees annex to Oldham Infirmary, new wing to St. Mary's Hospital, additional ward to Ross Memorial Hospital, Jubilee wing for out-patients to Southampton Infirmary, children's ward to Worthing Infirmary, Jubilee ward to Weymouth Royal Eye Infirmary, and a large extension of Waterford Union Hospital.

Amongst the projects sanctioned or commenced during the past year are a new ward for Addenbrooke's, fever hospital for Barnsley, convalescent home at Belfast, an accident ward for Boscombe Hospital, nurses' home for Bristol, new wing for Cardiff Infirmary, new branch of the Dreadnought, cottage hospital for Cheshunt, Dundee King's Cross Hospital, cottage hospital for Gorleston, residential students' college for Guy's, new infirmary for Halifax, new wing to Leed's Infirmary, Jubilee hospital for Mansfield, cottage hospital for Norwood, fever hospital for Rumworth, new wing for Sunderland Infirmary, Samaritan Free Hospital in Marylebone-road, eye hospital for Swansea, new infirmary for Stafford, and a new hospital for Woolwich and Plumstead.

The most noticeable point in these lists is the increase in the accommodation provided for infectious cases. Every large town and every country district will shortly have its fever hospital, and then we may hope to see the last of epidemics of smallpox and scarlet fever in England. Some 20 new hospitals opened and some 20 new hospitals commenced in one year is proof positive of the wide interest now taken in medical charities.

Miss Mary Harrison, of the Bayswater School of Cookery, 22, Westbourne-grove, is giving a series of lessons on cookery to the nurses at the "Dreadnought" Seamen's Hospital. Nurses unconnected with the hospital are allowed to attend, as are also ladies interested in the institution. The lessons, which include invalid and plain cooking, are much appreciated by the nurses and others.

The fifth annual sale of the Donegal Industrial Fund is now being held at 43, Wigmore-street, and some beautiful work is on view. The art embroideries are quite unique in their way, but the fine needlework of the napery and baby linen will appeal strongly to most women. That the Donegal peasantry should have so soon acquired these difficult arts shows the natural quickness of our Irish cousins, and we strongly commend the combined common-sense and sympathy which has started such industries in their midst. Those who buy their New Year presents at 43, Wigmore-street will perform a twofold charity; they will give their friends really well-made gifts, and they will help the poor women of Donegal.

Mr. Michelli, the able secretary of the Dreadnought Seamen's Hospital, has received the following letter dated Castleavery, Manitoba: "Having undertaken, in the event of my being proved mistaken as to the colour of a certain dog, to send to your hospital five dollars, and having this day been proved to be mistaken, I herewith enclose the above-mentioned sum, which will, I trust, be acceptable.—A. R. Binks." This letter is at once evidence of the world-wide fame of the Dreadnought Hospital, and of the attachment to the Old Country and its institutions which residents in our colonies so often manifest. If every wager henceforth to be won were similarly disposed of, the managers of our voluntary hospitals would soon be in funds.

The following were present at the meeting of the Hospitals Association on December 18: Mr. E. H. Lushington (treasurer of Guy's), in the chair; Mr. A. Wynter Blyth (medical officer of health for St. Marylebone), lecturer; Rev. C. H. Bowden (chaplain of Guy's), Dr. J. H. Gray, Miss N. Bickmore and Miss A. Gilliman (Evelina Hospital), Surgeon-Major J. Tree, Mr. P. Michelli (secretary Seamen's Hospital), Lieut.-Col. E. Montefiore, Miss R. Paget (Midwives' Institute), Mr. T. Ryan (secretary St. Mary's Hospital), Dr. J. Shaw (medical officer of health Rotherhithe), Dr. Steele and Mrs. Steele, Mrs. Suckling (lady superintendent Royal Hants County Hospital, Winchester), Dr. Wise (medical officer of health, Plumstead), and many sisters and nurses of Guy's Hospital, besides representatives of St. Thomas's, St. Bartholomew's, and University College Hospitals.

The influenza is still spreading in England and on the Continent, and it seems to be frequently followed by pneumonia and pleurisy. Though Spaniards persist in styling the present epidemic *dengue*, it is very generally thought that not a single case of real dengue has been seen yet. The Spanish doctors would easily have detected the existence of the dengue, as a few years ago that more serious epidemic raged in other districts, and particularly in Andalusia, with its characteristic symptoms of headaches, pains in the bones and joints, high fever and delirium, winding up with a skin eruption, itching, and blotches over the body. On the contrary, the present epidemic in Madrid seems in almost every case to be exclusively more or less a violent influenza, aggravated by nearly a whole month of very cold, dry, frosty weather. The common treatment used in Madrid among all classes is spare diet, purging, and repeated doses of magnesia. The medical papers say that more than 30,000 persons have been attacked in ten days.

The following vacancies are announced this week, the amount of the salary in each case being given after the name of the institution:

**Resident Medical Officers.**

Royal Berks Hospital. House Surgeon—£80, board, &c.
Birmingham General Hospital. Two Assistant Surgeons—board, &c.
Derbyshire General Infirmary. Assistant House Surgeon—board, &c.

**Non-Resident Medical Officers.**

Wolverhampton General Hospital. Two Honorary Gynaecologists.
Birmingham Lock Hospital. Third Officer.
Hospital for Sick Children, Great Ormond-street. Surgical Registrar—£40.
Chelmsford Union. Medical Officer—£140.
Dublin Mercer's Hospital. Physician.

Christmas gifts have been coming our way, and we have gladly forwarded a cheque for £1 from Mrs. Creighton Hale to the Seamen's Hospital, to be expended in pipes and tobacco for the patients. The clothing from Nurse Coates and from Blackheath (anonymous) we have forwarded to Charing Cross Hospital for the most needy of the patients there. *Truth* toys were distributed broadcast amongst sick children on Christmas morning, and we hope next year, by the kind help of our readers, to bring the wants of the adult sick more to the fore, and provide them each with a Christmas present.

The jury have decided that the two men who died at the works of the Leeds Forge Company on November 20, under strange circumstances, were suffocated from inhaling water gas, which was not at the time sufficiently odorised to be detected. The chief evidence tendered was that of Dr. Stevenson, the analyst for the Home Office, who had made an official examination and analysis of portions of the bodies. Dr. Stevenson said the result of his examinations was to show conclusively that both men died from poisoning by carbonic oxide, a constituent of water gas. It was the same deadly poison found in coal gas, in charcoal fumes, and in the fumes from iron-smelting furnaces. A number of deaths from water gas, it was stated, had lately been reported in America. The fact that the surgeons who conducted the post mortem were rendered unconscious has never been fully explained.

Lord Wolseley distributed ambulance certificates at Queen's Hall last week, and in the course of an excellent speech said that he was pleased to be at a meeting for the consideration of the art of alleviating human suffering from accidents met with in the streets or elsewhere. If there was any class in the world to whom the knowledge imparted by the association was valuable, it was to men who, like himself, were in scenes of danger, and where from hour to hour men needed help. Had he known as much years ago as he now knew, he could have rendered invaluable service. He had seen men bleeding to death beside him, not knowing how to help them. With regard to the kindred association of hospital nurses, he bore high testimony to the value of their work in the hospital camp in the Soudan, which was under the charge of Colonel Duncan during the war. The Queen had taken deep interest in the work of the association, and had sent an order to him that the decoration should be worn like other decorations won upon the battlefield. In conclusion, Lord Wolseley dwelt upon the value of women nurses in the Army instead of men, and illustrated this by his own personal experiences. He pointed out the heroic services which could be rendered by others than those who stormed a breach. We are glad to welcome Lord Wolseley to our side on this subject of women nurses for the Army.

The Rev. James G. Adderby issues a balance-sheet of the Strike Relief Fund collected and dispensed by those connected with the Christ Church Oxford Mission at the East India Docks. Over 15,000 dinners were given, and tickets for provisions and coals were distributed. The movement had the support of Mr. Sydney Buxton and Mrs. Charles Kingsley, so that funds were forthcoming, and much misery was spared the wives and children of the strikers.

The Parisian cab foot-warmers are not perfect, but we in England have none. An order of the Prefect of Police, who acted on the recommendation of the Board of Health, has been issued suppressing the foot-warmers heated with charcoal. Notwithstanding badly-jointed floors and doors, the cabs thus warmed have been proved to be very deadly. The Prefect of Police gives the owners the choice of having a ventilation tube to the foot-warmer to carry off the oxide of carbon, or to have it filled with hot water. It must be rather nice on getting, with damp feet, into a cab to feel the warmth of a foot-warmer.

The British Medical Association has been taking up the cause of Board school children, and has sent a deputation to the County Council on the subject. Dr. Savage stated that the object of the enquiry was to ascertain the proportion of children who were mentally or nervously unfitted for the ordinary course of education. Five thousand children had been examined, and the desire of the deputation was that permission should be given for the investigation of all schools. Any extra outlay would be more than compensated by the reduction in the criminal and vagrant classes. It was also thought that much of the nervous disorder that existed among scholars arose from defective eyesight. The subject was referred to the consideration of the School Committee.

The annual meeting of the Zenana Medical College was held on Tuesday evening, Dec. 10th, Rev. Canon Fleming in the chair. Prayer having been offered by the Rev. Joseph King, from Australia, and an anthem sung by the choir of Eccleston Church, who kindly gave their services during the evening, the hon. secretary read the report, which showed a large amount of work done both at home and abroad, as testified by letters received from many of the students. Canon Fleming, in his opening remarks, spoke of his connection with the college, of the pleasure it gave him to be present, and said that the report just read must have stirred deep thankfulness in many hearts. The Rev. F. E. Wigram, in moving the adoption of the report, said that he was personally acquainted with several of the ladies mentioned in it, and congratulated the committee on the prospect of receiving the two Syrian ladies nominated by Miss Proctor (a former student) for training at the college. W. S. Caine, Esq., M.P., in seconding the resolution, said that from his own observation he felt that the three great essentials for medical work amongst Indian women are a knowledge of nursing, of dispensing, and of maternity; that the lady who has mastered these is fitted for service in India, and that in whatever aspect he viewed the work of the Zenana Medical College he became more and more convinced of its value and importance. The second resolution was moved by the Rev. Jas. Sadler, of Amoy, who spoke of the needs of China and the immense value of a knowledge of simple remedies among the women of that country. Rev. J. Hiles Hitchens seconded the resolution, and ably supported the claims of the institution, and spoke of the high character of the instruction and training given. Rev. Richard Roberts, who occupied the chair after the unavoidable departure of Canon Fleming, supported the resolution. Dr. G. de G. Griffith read the financial statement, which was fairly satisfactory.

